# Triiodothyronine/Thyroxine Ratio as a Marker of Clinical Response to Levothyroxine Replacement in Patients With Hypothyroidism

**DOI:** 10.7759/cureus.58370

**Published:** 2024-04-16

**Authors:** Zaid M Hadi, Haider A Alidrisi, Abbas A Mansour

**Affiliations:** 1 Medicine, Faiha Specialized Diabetes, Endocrine, and Metabolism Center, Basrah, IRQ; 2 Diabetes and Endocrinology, Faiha Specialized Diabetes, Endocrine, and Metabolism Center, Basrah, IRQ; 3 Diabetes and Endocrinology, University of Basrah, College of Medicine, Basrah, IRQ

**Keywords:** levothyroxine, thyroid-stimulating hormone, t3/t4 ratio, triiodothyronine, thyroxine, hypothyroidism

## Abstract

Background: Hypothyroidism is one of the most common endocrine disorders with a simple therapy, that is levothyroxine (LT4). A normal thyroid-stimulating hormone (TSH) measurement is used as a marker of optimal replacement. But, many patients still have symptoms. Triiodothyronine (T3), thyroxine (T4), and their ratio may correlate with clinical improvement. The study aims to assess the T3/T4 ratio as a marker of clinical response in patients with hypothyroidism.

Method: A cross-sectional study was conducted from June to November 2022 at Faiha Specialized Diabetes, Endocrine, and Metabolism Center, in Basrah, southern Iraq. We included 48 adult patients with primary hypothyroidism on LT4 treatment only and TSH within the target reference range for at least within the last six months. Each patient was subjected to a questionnaire that was designed to capture hypothyroidism-related complaints in the form of a five-point Likert scale. Biochemical assessments were done with the measurement of TSH, T3, and T4.

Results: Despite having a normal TSH level, nearly all the patients had persistent and varying severity of clinical complaints of hypothyroidism. Tiredness, hair problems, weight gain, and cold intolerance were the most severely persistent symptoms. Patients with scores of two and more for weight gain, cold intolerance, and skin problems had significantly lower T3/T4 ratios (P = 0.04, 0.002, and 0.02, respectively), while in the remaining clinical symptoms, the T3/T4 ratio did not differ significantly.

Conclusion: A low T3/T4 ratio was significantly associated with resistant symptoms of hypothyroidism and may be used as a marker for treatment efficacy with TSH rather than TSH value alone.

## Introduction

Hypothyroidism is one of the most common endocrine disorders, with a greater burden of disease in women and the elderly [1]. The rate of hypothyroidism in countries with adequate iodine ranges from 1% to 2% [2,3], reaching 7% in those between the ages of 85 and 89 years [4]. An aging population is anticipated to lead to an increased prevalence of hypothyroidism in the lack of age-specific reference ranges for TSH. Women are around 10 times as likely as men to have hypothyroidism [2]. According to the latest studies based on large data derived from the Faiha Specialized Diabetes, Endocrine and Metabolism Center - FDEMC, the prevalence of hypothyroidism in Iraq was 12.5%, and women are five times more likely to get affected than men [5].

From asymptomatic disease to myxedema coma are a wide range of patient presentations that include lethargy, cramping or swollen muscles, unsteadiness, excess weight, hair loss, sensitivity to the cold, constipation, depressed mood, irregular or heavy periods, infertility, and bradycardia [6]. Nowadays, levothyroxine (LT4) monotherapy is the main treatment for hypothyroidism [7]. The recommended replacement dose of LT4 is 1.6 mcg/kg per day [8,9]. LT4 maintenance doses, once stable, often stay effective for patients until they are 60 to 70 years old.

Serial TSH measurements can be used to monitor the effectiveness of thyroid hormone replacement in individuals with an intact hypothalamic-pituitary axis. TSH values, however, do not change as quickly as blood thyroid hormone levels. As a result, the TSH level should only be assessed four weeks after a modification in the LT4 dosage. It may take up to eight weeks of treatment for the full rewards of thyroid hormone replacement on the TSH level to appear [10].

The only available marker for treatment monitoring in guidelines is TSH; however, many patients are still complaining, and some patients with hypothyroidism treated with LT4 have mentioned that their symptoms persisted despite reaching normal TSH levels [1,11,12], giving the idea that patients on levothyroxine therapy with normal thyrotropin are not necessarily euthyroid [13]. High serum T4/T3 ratios have been found in patients with hypothyroidism treated with LT4 [14]. Therefore, serum T4/T3 ratios may be used to monitor patients with unresolved symptoms in the presence of biochemical euthyroidism [15]. Other studies tried to evaluate the role of T3 or T4 measurement in LT4 therapy monitoring; however, no valid results were concluded [13,16].

This study was held to assess the clinical significance of thyroxine (T4), triiodothyronine (T3), and T3/T4 ratio as markers of clinical response to thyroxine treatment in patients with hypothyroidism.

## Materials and methods

A cross-sectional study was conducted from June to November 2022 at FDEMC in Basrah, southern Iraq. We included adult patients (18-65 years) with primary hypothyroidism on LT4 treatment only and TSH within the target reference range for at least within the last six months (confirmed by at least two TSH measurements through this period). Exclusion criteria included a history of diabetes mellitus, any cardiovascular disease, chronic liver disease, chronic kidney disease, celiac disease, psychological disorders, thyroid cancer or any other malignancies, and pregnancy. Furthermore, patients on any current medications that interfere with T4 and/or T3 measurements were excluded [17].

After a full assessment of the inclusion and exclusion criteria, 48 patients were included. Written informed consent was taken from each participant by the ethical standards of the FDEMC Research Committee, from which ethical approval was obtained (ref #45/13/22), and with the 1964 Declaration of Helsinki and its later amendments or comparable ethical standards.

Clinical assessment

The patients were subjected to a questionnaire designed to capture hypothyroidism-related clinical complaints. These complaints were tiredness, weight gain, cold intolerance, constipation, hair problems, skin problems, nail problems, hearing problems, voice problems, memory problems, appetite loss, dizziness, depression, and neuropathic pain. The patients were asked to scale these complaints in the form of a five-point Likert scale (from 0 to 4), in which zero meant no complaint and four meant the highest degree of complaint.

Biochemical assessment

From each patient, 3 mL of blood was drawn for the measurement of TSH, T4, and T3. The fully automated chemiluminescence immunoassay cobas e411 platform (Roche, Basel, Switzerland) was used for the measurement of serum TSH (normal range 0.27-4.2 µIU/mL), serum total thyroxine TT4 (normal range 5.1-14.1 µg/dL), and T3 (normal range 70-200 ng/dL).

Statistical analysis

The Statistical Package for the Social Sciences (SPSS), version 26.0 (IBM Corp., Armonk, NY) was used for data analysis. Categorical variables were summarized as numbers (N) and percentages (%). Continuous variables were summarized as mean ± standard deviation (M ± SD). For each patient, the T3/T4 ratio was calculated by dividing the T3 by the T4 level. The clinical complaints were grouped into two categories: the first with a score of one or less and the second with a score of two and more. The independent-student t-test (equal variance assumed) was used for the correlation between the T3/T4 ratio value and the clinical complaint categories. As a result of the cross-sectional study design, absence of a control group (single group study), and strict inclusion and exclusion criteria, we did not perform a study sample calculation. However, we included the effect size (d) and power analysis in the above comparisons. A P-value of <0.05 was defined as statistical significance for all the above comparisons.

## Results

Table 1 summarizes the general characteristics of the study patients. The mean age of the study patients was 42.2 ± 13.5 years, and 45 (93%) patients were females. The mean TSH for the included patients at the time of evaluation was 2.2 ± 1.3 μIU/mL.

**Table 1:** General characteristics of the study patients (N = 48) SD, standard deviation; TSH, thyroid-stimulating hormone; T4, thyroxine; T3, triiodothyronine, T3/T4 ratio, triiodothyronine level multiplied by thyroxine level in ng/µg.

Variable	Mean ± SD or N (%)	Reference range
Gender (female)	45 (93)	
Age (years)	42.2 ± 13.5	
TSH (μIU/mL)	2.2 ± 1.3	0.27-4.2 µIU/mL
T4 (μg/dL)	9.2 ± 3.3	5.1-14.1 µg/dL
T3 (ng/dL)	124.0 ± 30.9	70-200 ng/dL
T3/T4 ratio (ng/µg)	14.5 ± 4.7	-

**Figure 1:**
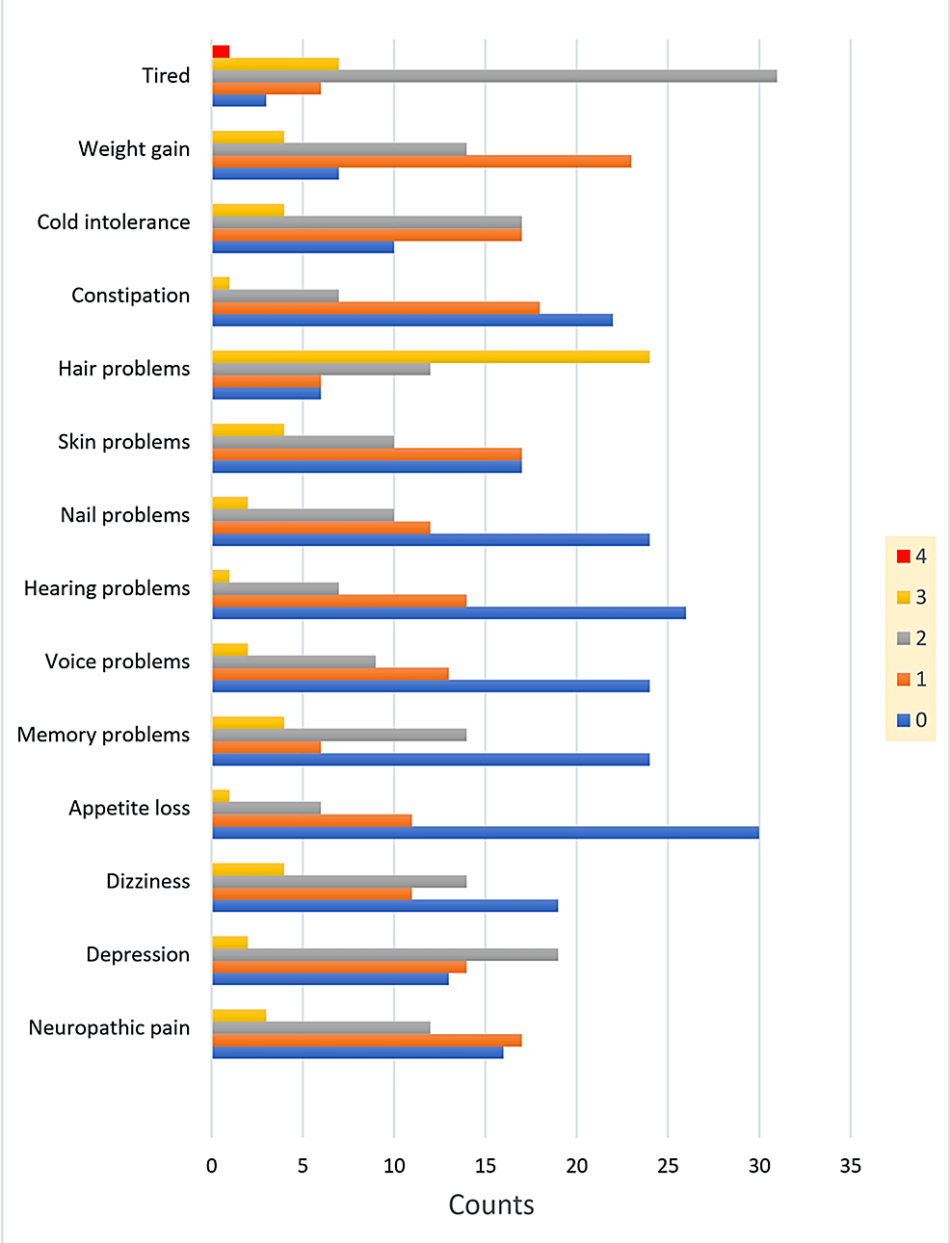
Patients' hypothyroidism clinical complaints score results. The patients were asked to scale hypothyroidism-related complaints in the form of tiredness, weight gain, cold intolerance, constipation, hair problems, skin problems, nail problems, hearing problems, voice problems, memory problems, appetite loss, dizziness, depression, and neuropathic pain using a five-point Likert scale, (from 0 to 4), in which zero meant no complaint and four meant the highest degree of the complaint.

The patients were asked to scale hypothyroidism-related complaints in the form of tiredness, weight gain, cold intolerance, constipation, hair problems, skin problems, nail problems, hearing problems, voice problems, memory problems, appetite loss, dizziness, depression, and neuropathic pain using a five-point Likert scale (from 0 to 4), in which zero meant no complaint and four meant the highest degree of the complaint. It is clearly seen in this study that despite adequate LT4 treatment and within the target TSH level, nearly all the patients had persistent and varying severity of clinical complaints of hypothyroidism, as shown in Figure 1. Clinical symptoms in the form of tiredness, hair problems, weight gain, and cold intolerance were the most highly scored persistent symptoms.

**Figure 2:**
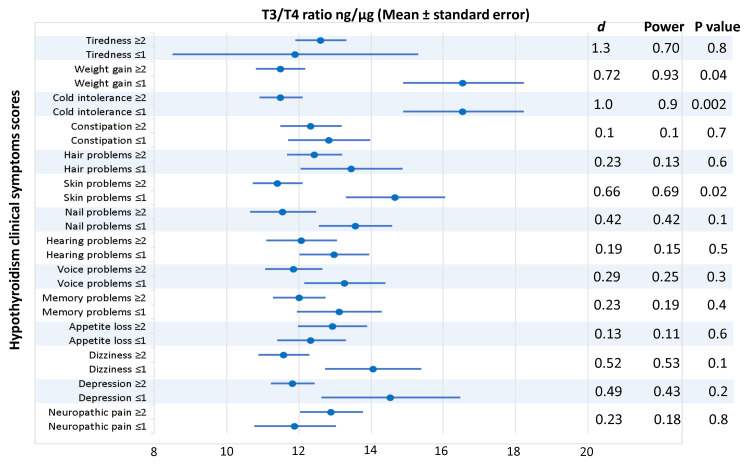
Correlations of the T3/T4 ratio and hypothyroidism clinical symptoms' scores. The patients were asked to scale hypothyroidism-related complaints in the form of tiredness, weight gain, cold intolerance, constipation, hair problems, skin problems, nail problems, hearing problems, voice problems, memory problems, appetite loss, dizziness, depression, and neuropathic pain using a five-point Likert scale from 0 to 4, in which zero meant no complaint and four meant the highest degree of the complaint. Based on the symptoms' scores, the patients were categorized into a group with a score of one or less and a group of two or more. The T3/T4 ratio (ng/µg) was compared between the two groups using the Independent-student t-test. T3/T4 ratio, triiodothyronine level multiplied by thyroxine level in ng/µg; *d, *effect size*.*

Based on the symptoms' scores, the patients were categorized into two groups, one with a score of one or less and another with a score of two or more. The T3/T4 ratio (ng/µg) was compared between the two groups. The T3/T4 ratio (ng/µg) was significantly low in the patients who scored two or more for weight gain, cold intolerance, and skin problems (P = 0.04, 0.002, and 0.02, respectively), while other patients' symptoms (tiredness, constipation, hair problems, nail problems, hearing problems, voice problems, memory problems, appetite loss, dizziness, depression, and neuropathic pain) did not correlate significantly with the T3/T4 ratio, as shown in Figure 2.

## Discussion

This study has clearly shown that despite having normal TSH, the patients with hypothyroidism had persistent various disease-related symptoms. We excluded hypothyroidism patients with other chronic diseases to avoid the overlap in symptomatology as possible. As a result of the subjective features of these symptoms, we tried to scale them and correlate their severity with the T3/T4 ratio which was significantly correlated with some of the symptoms' severity. Since the majority of the study patients were still complaining, the study did not perform an analysis for the T3/T4 ratio cutoff value for symptom persistence.

Given that T3 is the biologically active form of thyroid hormones, and that the thyrotropin assay has become the standard method for treating hypothyroid patients, it is reasonable to wonder whether treatment with levothyroxine (LT4) returned serum T3 levels to the normal range. This abruptly turned into a contentious issue [18].

T3 level in our study was extremely comparable to the mean of T3 levels in the normal population (124 ng/dL), while T4 was significantly higher than the reference range in the normal population (9.2 μg/dL). Similar findings were obtained from Mortoglou et al., who studied 1050 patients to investigate the significance of changes in T3, T4, and T3/T4 ratio in attaining euthyroidism in various thyroidal illnesses; they found that the mean T3 levels in the hypothyroid patients receiving thyroxine replacement were the same as those in the euthyroid group (119.85 vs. 124.47 ng/dL), but their T4 levels were significantly higher (9.11 vs. 7.99 g/dL).

The serum T3 levels in LT4-treated patients were low in numerous cross-sectional investigations evaluating the efficacy of therapy in patients with hypothyroidism [19]. However, one significant investigation evaluated serum T3 levels in 50 patients both before and after operative thyroidectomy and came to the conclusion that LT4 therapy, which normalizes serum thyrotropin, also returns serum T3 to preoperative values [20]. The reasons for the discrepancies surrounding serum T3 are still unclear.

Although all patients in our study achieved the target TSH level with LT4 therapy, they have remaining symptoms of varying severity, especially tiredness, hair problems, weight gain, and cold intolerance. In support of our findings, LT4-treated patients with normal serum thyrotropin display slower BMR, weigh around 10 pounds more, and report less physical activity and resistant tiredness, according to studies by Peterson et al. and Ridgway et al. [14,21]. Gullo et al. stated that an inability to sufficiently convert the levothyroxine they had consumed into T3 was the major cause of persistent symptoms [19]. Numerous factors contribute to that such as an inherited or acquired impairment in deiodinase activity, as well as abnormal thyroid hormone metabolism unrelated to deiodination [22]. Additionally, adequate treatment with LT4 did not associate with weight reduction according to a previous study. Instead, either the patient maintained the same weight or continued to gain more [11].

It is already established that patients with hypothyroidism receiving LT4 had a lower plasma T3/T4 ratio. However, it was comparable to that of the normal population in this study (14.5 ± 4.7). Furthermore, the present study shows strong evidence of an association between the low levels of T3/T4 ratio and weight gain, cold intolerance, and skin problems (P = 0.04, 0.002, 0.02), respectively. It is possible that some tissues are more susceptible to changes in the T3/T4 circulating ratio than others, as demonstrated by Salas-Lucia et al., who reported that each tissue has a unique fraction of free T3 produced through peripheral and local free T4 to free T3 conversion [23].

Escobar-Morreale et al. demonstrated in thyroidectomized rats that only the combination of T4+T3 therapy led to physiological T3 concentrations in all tissues [24]. Theoretically, a T4+T3 combination therapy may more closely resemble physiology, and free thyroid hormone concentrations would be more appropriate to give all tissues the best possible thyroid replacement. However, numerous clinical studies comparing T4 alone to T3+T4 regimens in hypothyroidism have not consistently demonstrated the superiority of combination therapy [25,26].

The study has limitations. First, the sample size being small might underpower the study results. Second is the subjective evaluation of the patients’ symptoms. Third, many of the patients’ symptoms might overlap with other associated diseases.

## Conclusions

Despite having a normal TSH level as an indicator of adequate LT4 replacement, the patients with hypothyroidism still had persistent symptoms. A low T3/T4 ratio was significantly associated with resistant symptoms of hypothyroidism in the form of weight gain, cold intolerance, and skin problems. The T3/T4 ratio may serve as a marker for treatment efficacy with TSH rather than TSH value alone.

## References

[1] Alidrisi HA, Musa AK, Mansour AA (2015). Clinical and social concerns in treated patients with primary hypothyroidism in Basrah: A cross sectional study. Am J Intern Med.

[2] Vanderpump MPJ (2011). The epidemiology of thyroid disease. Br Med Bull.

[3] Parle JV, Franklyn JA, Cross KW, Jones SC, Sheppard MC (1991). Prevalence and follow-up of abnormal thyrotrophin (TSH) concentrations in the elderly in the United Kingdom. Clin Endocrinol (Oxf).

[4] Gussekloo J, van Exel E, de Craen AJ, Meinders AE, Frölich M, Westendorp RG (2004). Thyroid status, disability and cognitive function, and survival in old age. JAMA.

[5] Mansour AA, Ali Alhamza AH, Abdullah Almomin AM (2020). SUN-418 patterns of thyroid disease in Basrah, Iraq. Retrospective study. J Endocr Soc.

[6] Li Y, Teng D, Ba J (2020). Efficacy and safety of long-term universal salt iodization on thyroid disorders: Epidemiological evidence from 31 provinces of Mainland China. Thyroid.

[7] Jonklaas J, Bianco AC, Bauer AJ (2014). Guidelines for the treatment of hypothyroidism: Prepared by the American Thyroid Association task force on thyroid hormone replacement. Thyroid.

[8] Ratanapornsompong G, Sriphrapradang C (2021). Appropriate dose of levothyroxine replacement therapy for hypothyroid obese patients. J Clin Transl Endocrinol.

[9] Borzì AM, Biondi A, Basile F, Vacante M (2020). Diagnosis and treatment of hypothyroidism in old people: A new old challenge. Wien Klin Wochenschr.

[10] Virili C, Giovanella L, Fallahi P, Antonelli A, Santaguida MG, Centanni M, Trimboli P (2018). Levothyroxine therapy: Changes of TSH levels by switching patients from tablet to liquid formulation. A systematic review and meta-analysis. Front Endocrinol (Lausanne).

[11] Alidrisi HA, Odhaib SA, Altemimi MT, Mansour AA (2021). Patterns of bodyweight changes in patients with hypothyroidism, a retrospective study from Basrah, Southern Iraq. Cureus.

[12] McAninch EA, Rajan KB, Miller CH, Bianco AC (2018). Systemic thyroid hormone status during levothyroxine therapy in hypothyroidism: A systematic review and meta-analysis. J Clin Endocrinol Metab.

[13] Ettleson MD, Bianco AC (2020). Individualized therapy for hypothyroidism: Is T4 enough for everyone?. J Clin Endocrinol Metab.

[14] Peterson SJ, McAninch EA, Bianco AC (2016). Is a normal TSH synonymous with "euthyroidism" in levothyroxine monotherapy?. J Clin Endocrinol Metab.

[15] Biondi B, Bartalena L, Chiovato L (2016). Recommendations for treatment of hypothyroidism with levothyroxine and levotriiodothyronine: A 2016 position statement of the Italian Society of Endocrinology and the Italian Thyroid Association. J Endocrinol Invest.

[16] Gomes-Lima C, Wartofsky L, Burman K (2019). Can reverse T3 assay be employed to guide T4 vs. T4/T3 therapy in hypothyroidism?. Front Endocrinol (Lausanne).

[17] Dong BJ (2000). How medications affect thyroid function. West J Med.

[18] Cooper DS (2008). Thyroxine monotherapy after thyroidectomy: Coming full circle. JAMA.

[19] Gullo D, Latina A, Frasca F, Le Moli R, Pellegriti G, Vigneri R (2011). Levothyroxine monotherapy cannot guarantee euthyroidism in all athyreotic patients. PLoS One.

[20] Jonklaas J, Davidson B, Bhagat S, Soldin SJ (2008). Triiodothyronine levels in athyreotic individuals during levothyroxine therapy. JAMA.

[21] Ridgway EC, Cooper DS, Walker H, Daniels GH, Chin WW, Myers G, Maloof F (1980). Therapy of primary hypothyroidism with L-triiodothyronine: discordant cardiac and pituitary responses. Clin Endocrinol (Oxf).

[22] Panicker V, Cluett C, Shields B (2008). A common variation in deiodinase 1 gene DIO1 is associated with the relative levels of free thyroxine and triiodothyronine. J Clin Endocrinol Metab.

[23] Salas-Lucia F, Bianco AC (2022). T3 levels and thyroid hormone signaling. Front Endocrinol (Lausanne).

[24] Escobar-Morreale HF, Del Rey FE, Obregón MJ, de Escobar GM (1996). Only the combined treatment with thyroxine and triiodothyronine ensures euthyroidism in all tissues of the thyroidectomized rat. Endocrinology.

[25] Saravanan P, Simmons DJ, Greenwood R, Peters TJ, Dayan CM (2005). Partial substitution of thyroxine (T4) with tri-iodothyronine in patients on T4 replacement therapy: Results of a large community-based randomized controlled trial. J Clin Endocrinol Metab.

[26] Escobar-Morreale HF, Botella-Carretero JI, Gómez-Bueno M, Galán JM, Barrios V, Sancho J (2005). Thyroid hormone replacement therapy in primary hypothyroidism: A randomized trial comparing L-thyroxine plus liothyronine with L-thyroxine alone. Ann Intern Med.

